# Pregnancy-specific anxiety and elective cesarean section in primiparas: A cohort study in China

**DOI:** 10.1371/journal.pone.0216870

**Published:** 2019-05-15

**Authors:** Yuanfang Sun, Kun Huang, Yabin Hu, Shuangqin Yan, Yeqing Xu, Peng Zhu, Fangbiao Tao

**Affiliations:** 1 Department of Maternal, Child and Adolescent Health, Anhui Medical University, Hefei, Anhui Province, China; 2 Anhui Provincial Key Laboratory of Population Health and Aristogenics, Hefei, Anhui Province, China; 3 Ma'anshan Maternal and Child Health Center, Ma'anshan, China; Chiba Daigaku, JAPAN

## Abstract

The purpose of this study was to investigate the association between pregnancy-specific anxiety and elective cesarean section, and identify the critical period in which pregnancy-specific anxiety will affect the elective cesarean section. Primiparous women in the 1^st^ trimester of pregnancy were invited to participate in the cohort. General information on maternal socio-demographic characteristics and environmental exposure were collected using questionnaires. Pregnancy-specific anxiety was assessed by using pregnancy-specific anxiety questionnaire in the 1^st^, 2^nd^ and 3^rd^ trimester, respectively. Delivery modes and pregnancy complications were abstracted from medical notes. Structural equation modeling (SEM) was adopted to examine the relationship between pregnancy-specific anxiety and elective cesarean section. Results indicated the overall elective cesarean section rate in this study was 45%. Among 1 874 pregnant women, 30.9% women experienced anxiety at least once during pregnancy, and 6.9% women suffered from anxiety in all three trimesters. Anxiety in the 2^nd^ trimester was a significant predictor for elective cesarean section. Young maternal age and low educational level had indirect effects on women’s choice of elective caesarean section through affecting pregnancy-specific anxiety. More attention should be paid to maternal psychological problems, and professional counseling needs to be strengthened to protect women from pregnancy-specific anxiety.

## Introduction

The rate of cesarean section (CS) has increased enormously in recent years [1–3]. In China, CS rate has increased from 46.2% in the WHO Global Survey of Maternal and Perinatal Health (WHOGS, 2004–08) to 47.6% in the WHO Multi-Country Survey of Maternal and Newborn Health (WHOMCS, 2010–11) [4]. The continuously increasing CS rate and the subsequent risk of maternal and neonatal morbidity have attracted considerable attentions [5–7]. Previous studies mainly focused on the influences of clinical factors on CS rates, such as pregnancy complications (e.g., gestational hypertension, preeclampsia, and gestational diabetes mellitus) and medical indications (e.g., fetal macrosomia, malpresentation, and placenta previa) [8–11].Growing research indicate that maternal mood disturbances in pregnancy may be an essential factor for the increasing CS rate by affecting maternal preference [12,13].

For most women, pregnancy is an inevitable and important period in their lives, which involves a series of physical and psychological changes [14]. These changes may cause great emotional fluctuations especially inprimiparous women. Pregnancy-specific anxiety, consisting of pregnancy-related worries and fears, is the most common emotional problem in pregnancy [15]. Pregnancy-specific anxiety has gradually been considered as a potential determinant that may affect women’s preference for CS. However, findings on the association between pregnancy-specific anxiety and CS are inconsistent. Several studies found that women with high anxiety levels were inclined to deliver babies via elective cesarean section (ECS) [12,16]. Another study indicated that pregnancy-related anxiety was associated with primary CS before and after controlling covariates [17]. On the contrary, some other studies presented that there was no relationship between pregnancy-specific anxiety and delivery modes [18,19]. These incompatible results occurred mainly due to the differences in research design, sample size, ethnicity, or the assessment of pregnancy-related anxiety. Also, most previous studies mainly focused on the influence of general anxiety in pregnancy [12,18,20], and did not measure pregnancy-specific anxiety separately in three trimesters of pregnancy [17–18]. In order to clarify the impact of the pregnancy-specific anxiety on childbirth, we adopted the pregnancy-specific anxiety questionnaire to assess pregnancy-specific anxiety of primiparas in the 1^st^, 2^nd^ and 3^rd^ trimester of pregnancy and tried to answer the following questions: (1) Does pregnancy-specific anxiety affect women’s decisions on ECS? (2) In which critical period during pregnancy may the pregnancy-specific anxiety be related to ECS?

## Materials and methods

Data used in this study was part of a broad project based on the Ma’anshan-Anhui Birth cohort (MABC) in China. Pregnant women in the 1^st^ trimester of pregnancy between May 2013 and September 2014 were recruited into the cohort with informed consents. After agreed to participate, they were asked to fill out the questionnaire surveys in the 1^st^, 2^nd^ and3^rd^ trimester of pregnancy, respectively, to collect basic demographic information and obstetric history of women. Their life patterns, environmental exposures, and pregnancy-specific anxiety in different trimesters of pregnancy were reported as well in these questionnaire surveys. Delivery modes, pregnancy complications and newborns’ birth characteristics were abstracted from the medical notes.

### Study populations

**Fig 1:**
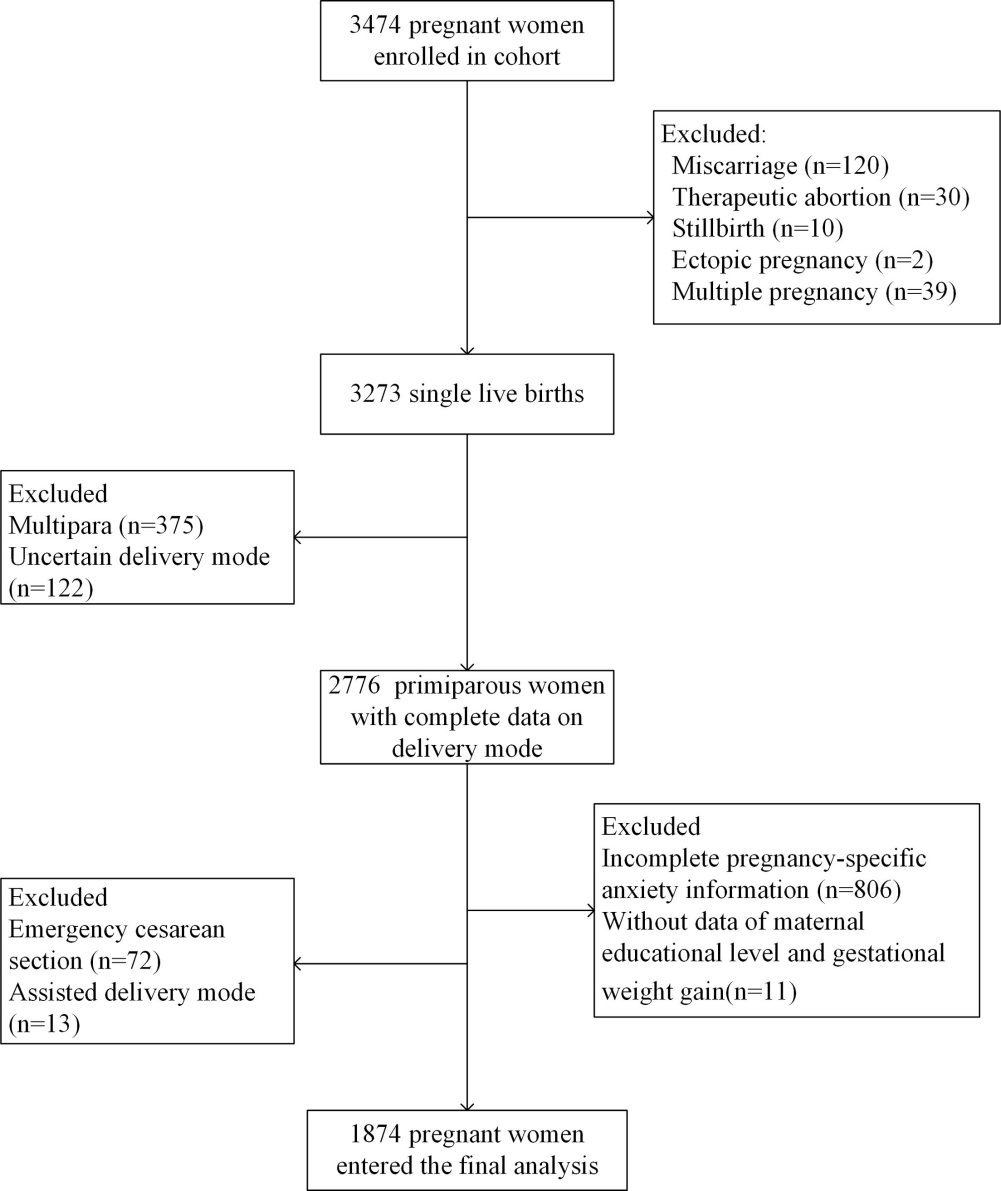
Enrollment flow chart of the birth cohort.

Pregnant women over 18 years old in their 1^st^ trimester were invited to participate in the cohort between May 2013 and September 2014. After written informed consents, a total of 3 474 eligible pregnant women were recruited in the study. After excluding participants with adverse pregnancy outcomes, multiple pregnancies, previous childbirths, assisted vaginal deliveries, emergency cesarean section, and those with incomplete data on delivery modes, pregnancy-specific anxiety, maternal educational level, and gestational weight gain, a total of 1 874 pregnant women were included in the final analysis (Fig 1).

Ethical approval for this research was approved by the ethical committee of Anhui Medical University (approval number 20131401).

### Participants’basic information

Pregnant women were asked to fill out a questionnaire when entering the cohort (in the 1^st^ trimester of pregnancy). It covered general information on maternal socio-demographic characteristics (including maternal age, educational level, and residence), environmental exposure (smoking and alcohol use) and pregnancy intention. Maternal weight and height were measured during this visit. The weight was regarded as the maternal pre-pregnancy weight and pre-pregnancy body mass index (BMI) was accordingly calculated. Body weight before delivery was collected in hospital where women had childbirth, and gestational weight gain was calculated by weight before delivery minus pre-pregnancy weight.

Pregnancy complications such as gestational hypertension, preeclampsia, and gestational diabetes mellitus (GDM) were collected from medical notes. GDM was defined as any degree of impaired glucose tolerance which was first discovered or diagnosed during pregnancy according to the American Diabetes Association (ADA) [21]. Gestational hypertension was defined as gestational systolic pressure ≥ 140 mmHg or diastolic blood pressure ≥90 mmHg, and preeclampsia was regarded as gestational systolic pressure ≥ 140 mmHg or diastolic blood pressure ≥ 90 mmHg with urinary protein ≥ 300mg in 24 hours or urinary protein ≥+ after 20 weeks of pregnancy [22]. We defined gestational hypertension and preeclampsia as hypertensive disorders in pregnancy.

### Assessment of pregnancy-specific anxiety

Pregnancy-specific anxiety was assessed by using a self-designed pregnancy-specific anxiety questionnaire in the 1^st^, 2^nd^ and 3^rd^ trimester, respectively. The questionnaire was designed for Chinese women and consisted of three subscales: anxiety for women’s own health (six items), anxiety for baby’s health (five items) and anxiety for childbirth (two items). The test-retest reliability coefficient of the total questionnaire, sub-score of anxiety for women’s own health, anxiety for baby’s health and anxiety for childbirth was 0.79, 0.67, 0.75, and 0.76, respectively. The Cronbach alpha coefficient of the total questionnaire, sub-score of anxiety for women’s own health, anxiety for baby’s health and anxiety for childbirth was 0.81, 0.64, 0.78, and 0.74, respectively. With confirmatory factor analysis, the validity indicators including goodness of fit index (GFI), normal fit index (NFI), relative fitting index (RFI), comparative fit index (CFI) and root mean square error of approximation (RMSEA) were 0.949, 0.897, 0.871, 0.904, and 0.070, respectively [23]. As far as we know, the pregnancy-specific anxiety questionnaire developed by our research team was the first instrument to assess pregnancy-specific anxiety in China and would provide an appropriate tool for future maternal psychosocial evaluation and intervention [24].

Women were asked to self-rate their anxiety status from 1–4 points varying from no worries, occasionally worried, often worried to always worried, and the total score was between 13 and 52. We followed previous research, i.e., Sun et al. 2016, to identify mothers with high level of pregnancy-specific anxiety, in which P73 criterion was used to determine the cut-off value (24) [25]. In P73 criterion, the top 27 percent of scores are treated as high level group, i.e., mothers with high level of pregnancy-specific anxiety. Women were determined to have high level of pregnancy-specific anxiety when they had total score ≥ 24. The cut-off of 24 was chosen based on the top 27 percent of the total scores on the pregnancy-specific anxiety questionnaire in all interviewees who participated in the development and reliability evaluation of the questionnaire. This cut-off selection was chosen because it was used in some computational algorithms for determining internal reliability indices [26]. Kelly [27] demonstrated that this number would provide a stable index of differences between groups with high and low level of certain tests.

### Definition of delivery mode

Delivery mode was abstracted from medical notes, which was recorded by the obstetrician at the time of delivery. It was classified into vaginal delivery, assisted vaginal delivery, ECS and emergency cesarean section. ECS was further defined as a planned cesarean section due to recognized medical indications and operation on maternal request without any obstetric indications [28]. Emergency cesarean section was defined as an unplanned operative delivery after onset of labor [29]. Due to the limited number of assisted vaginal delivery and emergency cesarean section, and given that both of them might result from certain obstetric conditions, these two delivery modes were excluded from the final analysis.

### Statistical analysis

Data was analyzed using SPSS (version 16.0) and Amos (version 17.0) software packages. Continuous variables were expressed by means and standard deviations, and categorical variables were shown as frequencies. Independent-samples *t* tests and chi-square tests were used to compare the maternal socio-demographic characteristics and the scores of pregnancy-specific anxiety in the 1^st^, 2^nd^ and 3^rd^ trimesters between women with vaginal delivery and women with ECS. A *p* value below 0.05 was considered to be statistically significant.

**Fig 2:**
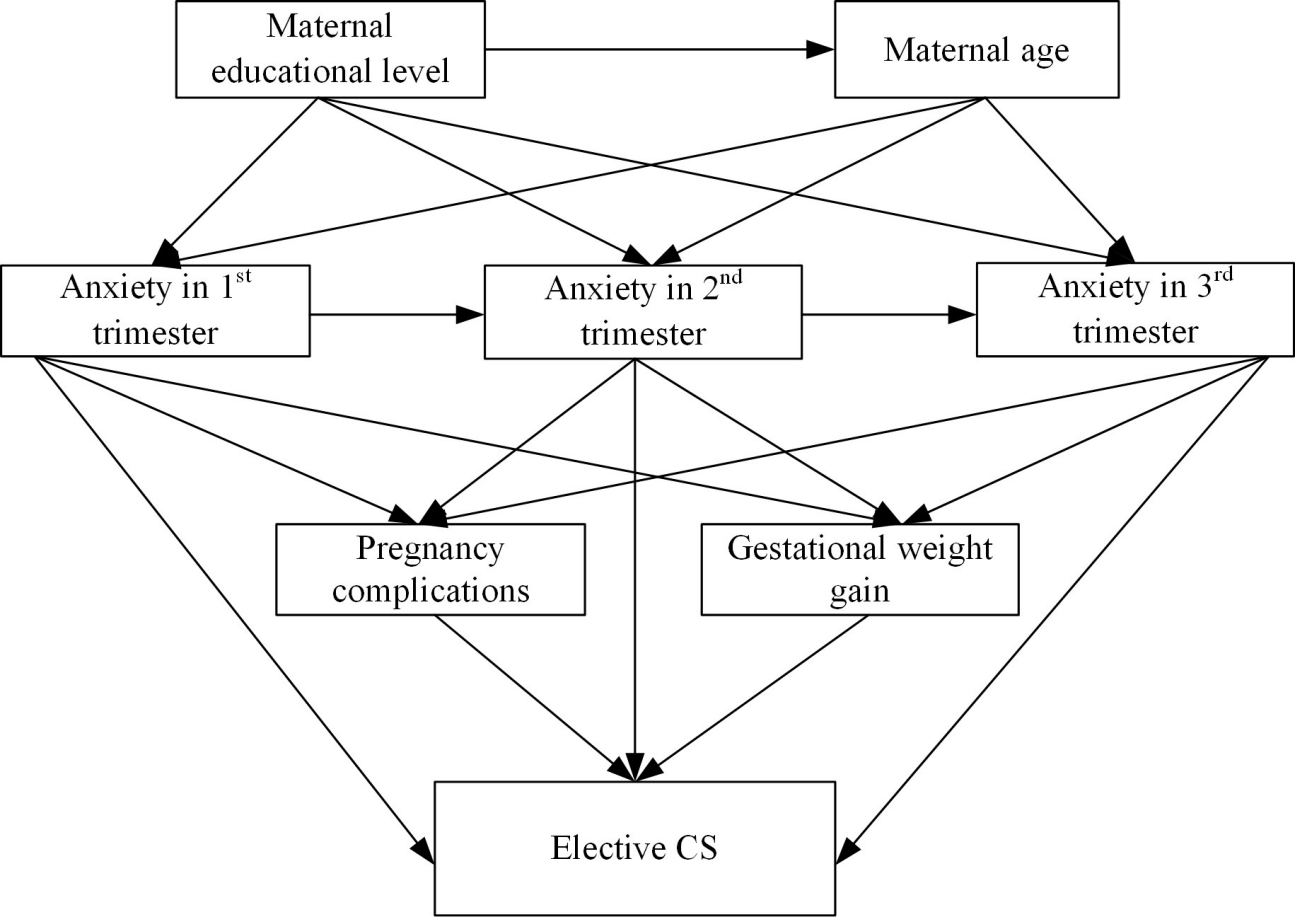
Structural equation modeling showing relationship about pregnancy-specific anxiety and elective caesarean sections.

In order to explore the association between pregnancy-specific anxiety different periods of pregnancy and elective cesarean section, avoid the multi-collinearity issues among potential confounders, and explain their direct and indirect effects on the choice of delivery mode, the structural equation modeling (SEM) was adopted for the analysis. In light of previous studies [17,30] and data availability in our study, we used maternal age, maternal educational level, pregnancy-specific anxiety (by using total scores), pregnancy complications and gestational weight gain to build the structural equation model (Fig 2). The hypothesis was supported at the 0.05 level.

## Results

In 2 776 primiparous women with complete data on delivery mode, the overall CS rate was 47.6% (1 321/ 2 776), and ECS rate was 45.0% (1 249/2776).

We compared the characteristics between women included in final analysis and those excluded due to incomplete pregnancy-specific anxiety information. There was no significant difference in socio-demographic characteristics between the two groups. But the excluded women had a significantly higher prevalence of pregnancy complications than the included women (*P*<0.05).

After excluding incomplete data on necessary variables, the socio-demographic characteristics in final participant samples are summarized in Table 1. There was significant difference in maternal age and pre-pregnancy BMI (*P*<0.01) between women with different delivery modes. Older women and women with higher pre-pregnancy BMI were more likely to choose ECS. Also women with ECS had more gestational weight gain, more frequencies of smoking and higher risk to experience gestational diabetes mellitus and hypertension disorders in pregnancy.

**Table 1:** Maternal basic characteristics in relation to delivery modes.

Characteristics	Total (n = 1 874)	Women with vaginal delivery (n = 1 020)	Women with ECS (n = 854)
Maternal age (years)^**^, mean±SD	26.13±3.10	25.80±2.78	26.53±3.40
Maternal educational level (years), mean±SD	13.61±3.03	13.64±3.08	13.58±2.98
Urban registration, n(%)	1125(60.0)	592(58.0)	533(62.4)
Pre-pregnancy BMI (kg/m^2^)^**^, mean±SD	20.66±2.68	17.10±4.80	21.12±2.95
Smoking during pregnancy, n(%)	80(4.3)	32(3.1)	48(5.6)
Alcohol useduring pregnancy, n(%)	156(8.3)	76(7.4)	80(9.4)
Gestational diabetes mellitus^**^, n(%)	156(8.3)	76(7.4)	80(9.4)
Hypertensive disorders^**^, n(%)	98(5.2)	30(2.9)	68(8.0)
Gestational weight gain (kg), mean±SD	17.56±5.01	20.28±2.36	21.12±2.95
Pregnancy intention, n(%)^*^			
Well-prepared pregnancy	481(25.7)	242(23.7)	239(28.0)
Unintended pregnancy	1393(74.3)	778(76.3)	615(72.0)

^*^P < 0.05,

^**^P < 0.001.

Abbreviations: SD = standard deviation; BMI = body mass index; CS = cesarean section.

The total scores of anxiety were 20.40±4.83, 19.75±4.53 and 18.86±4.21in the 1^st^, 2^nd^ and 3^rd^ trimester, respectively. During the 1^st^ trimester, women with vaginal delivery had high sub-scores of anxiety for their own health (*P*<0.05). In the 2^nd^ trimester, women with ECS had higher total scores (*P*<0.05), along with higher sub-scores of anxiety for baby’s health (*P*<0.01). And in the 3^rd^ trimester, women with ECS had higher sub-scores of anxiety for childbirth (*P*<0.05) (Table 2).

**Table 2:** The distribution of pregnancy-specific anxiety in different gestational period in relation to delivery modes (mean±SD).

Gestational period	Pregnancy-specific anxiety scores	Total (n = 1874)	Women with vaginal delivery (n = 1 020)	Women with ECS (n = 854)
1^st^ trimester	Total score	20.41±4.83	20.37±4.77	20.43±4.90
	Sub-scores of anxiety for women’s own health^*^	7.51±1.75	7.60±1.81	7.42±1.67
	Sub-scores of anxiety for baby’s health	9.13±2.86	9.03±2.85	9.24±2.87
	Sub-scores of anxiety for childbirth	3.76±1.41	3.74±1.37	3.78±1.46
2^nd^ trimester	Total score^*^	19.75±4.53	19.50±4.27	20.04±4.81
	Sub-scores of anxiety for women’s own health	7.52±1.76	7.47±1.67	7.59±1.86
	Sub-scores of anxiety for baby’s health^**^	8.43±2.44	8.30±2.31	8.60±2.58
	Sub-scores of anxiety for childbirth	3.79±1.39	3.74±1.34	3.85±1.44
3^rd^ trimester	Total score	18.86±4.21	18.79±4.09	18.94±4.34
	Sub-scores of anxiety for women’s own health	7.31±2.28	7.33±1.66	7.29±1.65
	Sub-scores of anxiety for baby’s health	7.82±2.28	7.80±2.28	7.86±2.27
	Sub-scores of anxiety for childbirth^*^	3.72±1.25	3.66±1.17	3.80±1.34

^*^P < 0.05,

^**^P < 0.001.

Abbreviations:SD = standard deviation; CS = cesarean section.

### The relationship between pregnancy-specific anxiety and ECS

Different levels of pregnancy-specific anxiety in the 1^st^, 2^nd^ and 3^rd^ trimesters are presented in Table 3. There were 30.9% (579/1 874) of women who experienced anxiety at least in one trimester, and 6.9% (130/1 874) suffered from anxiety through the whole span of pregnancy. In women with anxieties, there was a non-significant tendency that more proportion of women chose ECS with the increasing number of trimesters when they experienced pregnancy-specific anxiety (*P* = 0.139).

**Table 3:** Different level of pregnancy-specific anxiety in relation to delivery modes.

Different levels of pregnancy-specific anxiety	Total (n = 1 874)	Women with vaginal delivery (n = 1 020)	Women with ECS (n = 854)
Without anxiety	1295	712(55.0)	583(45.0)
High anxiety level in one trimester	287	165(57.5)	122(42.5)
High anxiety level in two trimester	162	83(51.2)	79(48.8)
High anxiety level in three trimester	130	60(46.2)	70(53.8)

Abbreviation: CS = cesarean section.

### The association of pregnancy-specific anxiety with delivery modes

The structural equation model is shown in Fig 2. The values of reliability metrics showed that the model fit was acceptable (Chi-square = 181.770, *P*<0.001; GFI = 0.977; NFI = 0.939; CFI = 0.941; RMSEA = 0.108). In the model, pregnancy complications, gestational weight gain, and total scores of anxiety in the 2^nd^ trimester were found to be significantly associated with ECS. The estimates of the interrelationships among these factors in the model and their significance values are described in Table 4.

**Table 4:** Standardized estimates of correlated variables in structural equation model.

Correlated variables	estimate	*p*-value
Maternal educational level → Maternal age	0.258	<0.001
Maternal educational level → Anxiety in 1^st^ trimester	-0.065	0.006
Maternal educational level → Anxiety in 2^nd^ trimester	-0.002	0.933
Maternal educational level → Anxiety in 3^rd^ trimester	0.001	0.939
Maternal age → Anxiety in 1^st^ trimester	-0.088	<0.001
Maternal age → Anxiety in 2^nd^ trimester	-0.007	0.691
Maternal age → Anxiety in 3^rd^ trimester	0.000	0.992
Anxiety in 1^st^ trimester → Anxiety in 2^nd^ trimester	0.639	<0.001
Anxiety in 2^nd^ trimester → Anxiety in 3^rd^ trimester	0.753	<0.001
Anxiety in 1^st^ trimester → Pregnancy complications	-0.023	0.448
Anxiety in 1^st^ trimester → Gestational weight gain	0.031	0.299
Anxiety in 2^nd^ trimester → Pregnancy complications	0.056	0.162
Anxiety in 2^nd^ trimester → Gestational weight gain	0.052	0.194
Anxiety in 3^rd^ trimester → Pregnancy complications	-0.023	0.519
Anxiety in 3^rd^ trimester → Gestational weight gain	0.015	0.665
Anxiety in 1^st^ trimester →ECS	-0.043	0.150
Anxiety in 2^nd^ trimester →ECS	0.111	0.005
Anxiety in 3^rd^ trimester →ECS	-0.048	0.168
Pregnancy complications →ECS	0.109	<0.001
Gestational weight gain →ECS	0.099	<0.001

Abbreviation: CS = cesarean section.

Pregnancy-specific anxiety in three trimesters significantly, positively, and progressively correlated with each other. Women with high scores of anxiety in the 1^st^ trimester were more likely to have high scores in the 2^nd^ trimester, and scores in 2^nd^ trimester were likely related with those in the 3^rd^ trimester. Younger women and women with lower educational level were more likely to have higher scores of anxiety in the 1^st^ trimester. No significant relationships were found between maternal age, maternal educational level and anxiety in the 2^nd^ and 3^rd^ trimesters.

In this study, high scores of anxiety in the 2^nd^ trimester, along with pregnancy complications and more gestational weight gain were independently and directly associated with the maternal choice of ECS. An indirect effect was observed as maternal educational level, maternal age, anxiety in the 1^st^ trimester, anxiety in the 2^nd^ trimester and choice of ECS. Women with lower educational level were more likely to be younger, younger women were more likely to have high anxiety level in 1^st^ and 2^nd^ trimesters, and thus were more inclined to choose ECS.

## Discussion

This study reported an overall CS rate was 47.6%, and the prevalence of ECS was 45.0% in primipaous women. High level of anxiety in the 2^nd^ trimester was directly related with women’s choice of ECS. An indirect effect was observed via women’s lower educational level, younger age, higher anxiety level in 1^st^ and 2^nd^ trimester which ultimately resulted in the higher likelihood of choosing ECS.

The reported overall CS rate was similar to the overall annual CS rate in China reported by WHO (47.6% in 2010–12) [4], but was lower than the rate reported in Brazil (56% in 2014) [31]. The ECS rate in this study was much higher than the rate in southeast China (20%) [32].

In this study, 30.9% of women experienced anxiety at least in one trimester of pregnancy, and 6.9% women suffered from anxiety throughout their pregnancy. The prevalence was similar to the overall rate of prenatal anxiety (28.0%) in the All Our Babies pregnancy cohort study, which included women with a gestational age less than 24 weeks [33]. In the current study, anxiety of women’s own health in early pregnancy, worries of baby’s health during gestation and anxiety for childbirth around delivery mirrored the general mental state of pregnant women. In terms of total scores, significant difference between vaginal delivery and ECS only existed in the 2^nd^ trimester of pregnancy.

Our findings demonstrated that anxiety in the 2^nd^ trimester was a significant risk factor for the choice of ECS. Although some previous research did not confirm the association between pregnancy-specific anxiety and delivery types [18,34], there were also several previous findings in line with the current study [35,36]. In the Amsterdam Born Children and their Development (ABCD) Study and the Heidelberg Peripartum Study, pregnant women, with gestational ages more than 24 weeks, who experienced pregnancy-specific anxiety were more likely to choose ECS [17,34]. A supporting result was reported by Andersson et al [36] indicating a significant association between antenatal anxiety and ECS by using the Primary Care Evaluation of Mental Disorders system to assess the anxiety during pregnancy. Some other studies demonstrated that anxiety during pregnancy and fear of childbirth had main impact on women’s preference for ECS [37–39].

The results from the structural equation modeling showed that anxiety in the 2^nd^ trimester directly related with women’s preference for ECS; and maternal age and educational level had indirect effect on delivery mode via anxiety in 1^st^ and 2^nd^ trimester. The findings suggested that high level of anxiety in the 2^nd^ trimester independently caused the preference for ECS, not via the commonly-believed pregnancy obstetric-related conditions. The results were similar to previous studies which suggested that anxiety in pregnancy would increase the risk for ECS [17,36], women with lower educational level would have worse pregnancy-specific anxiety status [17,40], and younger women would experience more severe anxiety during pregnancy [41]. It also highlighted that younger women and poorly-educated pregnant women were more vulnerable to pregnancy-specific anxiety. It was noted that women excluded had significantly higher prevalence of pregnancy complications than those included in the analysis. Some studies claimed that maternal anxiety would increase gestational weight gain, hypertensive disorders of pregnancy, and gestational diabetes mellitus [15,42–44]. It is possible that the exclusion had led to a bias since pregnancy complications and/or might be linked to pregnancy anxiety which in turn might be associated with having ECS. We had tested the hypothesis in SEM and found no significant associations between pregnancy-specific anxiety in three trimesters and pregnancy complications or gestational weight gain. Due to the incomplete data in the excluded women, however, we could not verify the association among this group. The bias might indeed exist and further research with a larger study sample is needed.

The mechanism by which pregnancy-specific anxiety affected delivery mode remains unclear. Studies had shown that pregnancy-specific anxiety and fear were mostly due to an incorrect understanding by pregnant mothers and uncertainty of their ability to deliver naturally [45]. Previous studies pointed out that women with pregnancy-specific anxiety, especially the fear of childbirth, were more easily to be in doubt of themselves and the obstetric staff, and more likely to feel incapable of giving birth naturally [46–47]. Women with anxiety and fear were more likely to feel insufficient support from their family members during labor and loss of control, and were more likely to worry about fetal injury/death [13,48,49]. Anxiety during pregnancy may aggravate maternal fear of childbirth and lead to their refusal to deliver vaginally [50,51]. A Swedish study confirmed that women with fear of childbirth were more likely to fear at the beginning of labor, thus would tend to choose CS [52]. Also, pregnancy anxiety and fear of childbirth were significant emotional manifestations during pregnancy and could affect labor [53]. Feeling anxious and worried would cause the biological stress response, which could activate neuroendocrine response involving the hypothalamic-pituitary-adrenal (HPA) axis [54]. Some studies argued that when women experience anxiety and fear, plasma catecholamines concentrations and cortisol concentration would elevate, and high levels of stress hormones would attenuate uterine contraction in both animals and humans [55–57], which is necessary for normal onset of labor. It is speculated that women with delayed onset of labor might ask for a cesarean delivery. Further in-depth studies are needed to explore the mechanism underlying the association between demographic characteristics, psychological features and delivery modes.

There are several strengths of our research. Firstly, most previous studies used general anxiety scales to evaluate maternal anxiety during pregnancy, and it is believed that general anxiety could explain only 8% to 10% for the variance for the anxiety in the 1^st^ trimester and 2^nd^ trimester, respectively [14]. We assessed pregnancy specific anxiety by the instrument particularly designed for Chinese women. Secondly, several previous studies had reported that primipara were more intensively associated with high pregnancy-specific anxiety level [15,58]. Thus in this cohort study, we only focused on primiparous women, thereby limiting the impact of parity on the observed findings. Finally, the prospective cohort study allowed us to accurately collect and control potential confounding factors during the natural progress of pregnancy and childbirth, leading to reduction in recall bias.

Some limitations should be considered when interpreting our results. Firstly, due to the strong collinearity among the independent variables, we adopted structural equation modeling to identify the relationship between pregnancy-specific anxiety and delivery mode. On account of the binary requirement of categorical variables, we classified delivery mode into vaginal delivery and ECS and did not distinguish between ECS with and without medical indications. Women with medical indications might choose CS regardless of pregnancy-specific anxiety. Secondly, we did not consider other emotional problems such as depression and anxiety disorders. These emotional problems and the probable use of antidepressants might also have some impacts on the relationship between pregnancy-specific anxiety and ECS.

## Conclusion

Pregnancy-specific anxiety, especially in the 2^nd^ trimester, independently and directly resulted in primiparous women’s vulnerability to ECS. Young pregnant women and women with low literacy level were potentially at high risk to have pregnancy-specific anxiety. Prenatal counseling from medical staffs with appropriate approaches should be strengthened to protect women from pregnancy-specific anxiety, and the 2^nd^ trimester of pregnancy could be the key period for such interventions. Young women and poorly-educated women should be the focus groups to perform the counseling.

## Supporting information

S1 Dataset (SAV)
